# Regulatory mechanisms of B cell responses and the implication in B cell-related diseases

**DOI:** 10.1186/s12929-019-0558-1

**Published:** 2019-09-01

**Authors:** Dong-Yan Tsai, Kuo-Hsuan Hung, Chia-Wei Chang, Kuo-I Lin

**Affiliations:** 1 Genomics Research Center, Academia Sinica, 128 Academia Road, Sec. 2, Nankang Dist, Taipei, 115 Taiwan; 2 Graduate Institute of Immunology, College of Medicine, National Taiwan University, Taipei, 110 Taiwan

**Keywords:** B cell, Antibody, Plasma cell, Transcription factor, microRNA, Epigenetic regulation, B cell malignancy, Autoimmune disease

## Abstract

Terminally differentiated B cell, the plasma cell, is the sole cell type capable of producing antibodies in our body. Over the past 30 years, the identification of many key molecules controlling B cell activation and differentiation has elucidated the molecular pathways for generating antibody-producing plasma cells. Several types of regulation modulating the functions of the important key molecules in B cell activation and differentiation add other layers of complexity in shaping B cell responses following antigen exposure in the absence or presence of T cell help. Further understanding of the mechanisms contributing to the proper activation and differentiation of B cells into antibody-secreting plasma cells may enable us to develop new strategies for managing antibody humoral responses during health and disease. Herein, we reviewed the effect of different types of regulation, including transcriptional regulation, post-transcriptional regulation and epigenetic regulation, on B cell activation, and on mounting memory B cell and antibody responses. We also discussed the link between the dysregulation of the abovementioned regulatory mechanisms and B cell-related disorders.

## Background

### The life journey of B cells – from development to activation and differentiation

B cell development begins in the fetal liver and continues in hematopoietic stem cells (HSCs) in the bone marrow where the stromal cells provide cytokines and chemokines, such as C-X-C motif chemokine 12 (CXCL12) and interleukin (IL)-7, for early stage B cell development [1]. The signals from the stromal cells allow HSCs to differentiate into common lymphoid progenitor cells (CLPs), which express c-kit and IL-7 receptors to provide the survival and proliferation signals for CLPs once they encounter the ligands. Upon expression of the transcription factors, E2A and early B-cell factor (EBF), CLPs further develop into pro-B cells [2]. Starting from pro-B cells, B cells in the bone marrow experience a sequential genetic rearrangement of heavy-chain and light-chain immunoglobulin genes, the V(D)J recombination, resulting in the generation of the IgM-expressing immature B cells [3]. The immature B cells migrate from the bone marrow to the spleen, where they further differentiate into T1 and T2 stages. B cells finally become mature B cells that co-express IgD and IgM, after which they wait to be activated by foreign antigens [4].

For activation and differentiation into antibody-secreting plasma cells, mature B cells in the periphery lymphoid organs require two signals. The first signal is derived from antigen-coupled B cell receptors (BCRs), and the second signal can be delivered in a T cell-dependent (TD) or T cell-independent (TI) manner. TI antigens, such as lipopolysaccharides (LPS) and glycolipids, mostly give rise to short-lived plasma cells that produce low-affinity antibodies. TD responses, initiated by antigen encounter and interaction with follicular helper T (Tfh) cells [5], allow B cells to either quickly become short-lived plasma cells or enter the germinal center (GC) to differentiate into plasma cells or memory B cells with higher affinity toward the antigens. The GC can be polarized into the dark zone, where B cells undergo somatic hypermutation (SHM) at the variable regions of the BCR genes and clonal expansion, or the light zone, where B cells go through affinity maturation via interaction with Tfh cells and follicular dendritic cells (FDCs) to select B cell clones with high affinity BCRs [6]. Tfh cells produce the CD40 ligand for maintaining B cell survival, and IL-21 for promoting cell proliferation and differentiation [7]. In GC B cells, class switch recombination (CSR) that changes the constant region of the immunoglobulin from one isotype to another also occurs. GC B cells that are not positively selected by FDCs are eliminated by apoptosis, while the selected B cells may re-enter the dark zone to re-evolve BCRs with better affinity. The GC reaction allows B cells with high affinity receptors to further differentiate into plasma cells or memory B cells [8]. The GC-derived plasma cells circulate to the bone marrow and secrete antigen-specific antibodies to become long-lived plasma cells that provide long-term protection against specific antigens [9].

## Main text

### Transcriptional network in mature B cells and plasma cells

B cell differentiation is tightly controlled by a transcription regulation network. It involves the coordination of several transcription factors to promote the expression of antibody-secretion and plasma cell-related genes, and downregulate the B cell identity genes. B lymphocyte-induced maturation protein-1 (Blimp-1) is a critical transcription regulator of plasma cell formation, which mainly functions as a transcription repressor [10]. Deficiency in Blimp-1, encoded by the PR domain zinc finger protein 1 (*Prdm1*) gene, in mice impaired plasma cell differentiation, but did not affect B cell development [11]. Blimp-1 is expressed at low levels during the plasmablast stage, and at high levels in mature plasma cells [12]. Mechanistically, Blimp-1 represses the genes important for B cell identity, such as paired box protein 5 (*PAX5*), B-cell lymphoma-6 (*BCL6*) and BTB domain and CNC Homolog 2 (*BACH2*) [13, 14], and induces the activation of interferon regulatory factor 4 (*IRF4*) and X-Box Binding Protein 1 (*XBP-1*) [15, 16], suggesting that it has a multifunctional role in transcription regulation. PAX5 is expressed throughout the early B cell developmental stages and in mature B cells, and is crucial for the maintenance of the identity of the B cell lineage [17]. Furthermore, it regulates the expression of BCR component genes, such as *CD19*, *CD21* and *IgH*, and other transcription factors important for B cells, like *IRF4*, *IRF8*, *BACH2*, Ikaros family zinc finger protein 3 (*IKZF3*) and *PRDM1* [18]. After the B cells are activated and enter the GC B-cell stage in the secondary lymphoid organs, BCL6 and BACH2 expression begins. Upregulation of BCL6 is critical for the formation of GC and the prevention of plasma cell differentiation [19, 20]. Signaling through IL-21 receptor in proliferating GC B cells sustains the expression of BCL6 [21]. BACH2 is expressed in the pro-B to mature B cell stages, and is absent in plasma cells. Loss of BACH2 causes the lack of GC and *Aicda,* encoding activation-induced cytidine deaminase (AID), which is critical for SHM and CSR [22]. Both BCL6 and BACH2 suppress the expression of *PRDM1* [23, 24]. In addition to Blimp-1, plasma cell formation requires IRF4, which represses *Bcl6*, therefore inducing Blimp-1 expression [25, 26]. Loss of IRF4 leads to impaired antibody production [27]. XBP-1 functions as a transcription regulator that is essential for Ig secretion and remodeling of the endoplasmic reticulum in plasma cells [28]. Absence of Blimp-1 causes impaired expression of XBP-1 and its downstream genes, suggesting that Blimp-1 is necessary for XBP-1 induction [29].

### miRNA in B cell activation and differentiation

MicroRNAs (miRNAs) are small non-coding RNAs containing approximately 22–23 nucleotides (nts) in length that play important roles in post-transcriptional regulation in several biological processes, including apoptosis, cell proliferation, cell cycle, cell differentiation, hematopoiesis and cancer [30]. Studies on miRNA functions have revealed that one miRNA can specifically target hundreds of different mRNAs, and every single mRNA can be regulated by several different miRNAs [31, 32].

More than 1000 miRNAs have been identified in the human genome, which target about 60% of the human protein-encoding genes [33]. More than 100 different miRNAs are expressed by the immune system cells [34–36]. MiRNAs have the potential to broadly influence the molecular pathways that control the development and functions of innate and adaptive immune responses. Global miRNA expression profiling in various B cell stages has been reported [37]. An atlas of human mature B cell miRNAs (“miRNome”) was constructed with mature B cell line-specific short-RNA libraries coupled with low throughput sequencing [36]. Furthermore, miRNA array has been extensively used to identify miRNA expression profiles. For example, miRNA array profiling of CD5^+^-activated and CD5^−^-resting B cells from human peripheral blood and tonsils revealed that 34 miRNAs were enriched in CD5^+^-activated B cells, and eight of them, including miR-323, miR-138, miR-9*, miR-211, miR-129, miR-373, miR-135a and miR-184, were highly expressed miRNAs capable of co-targeting *ZEB1* and *TP53* [38]. The importance of miRNAs in B cell lineage was emphasized by a study on a mouse gene knockout model in which *Dicer*, encoding a key enzyme responsible for the generation of miRNAs from their precursors [39], is deleted in a B cell-specific manner. B cell-specific deletion of *Dicer* exhibited a developmental block at the pro-B to pre-B stages and revealed that miRNAs may have a role in controlling V(D)J recombination for generating antibody diversity in the early stage of B cell development [40].

**Fig. 1:**
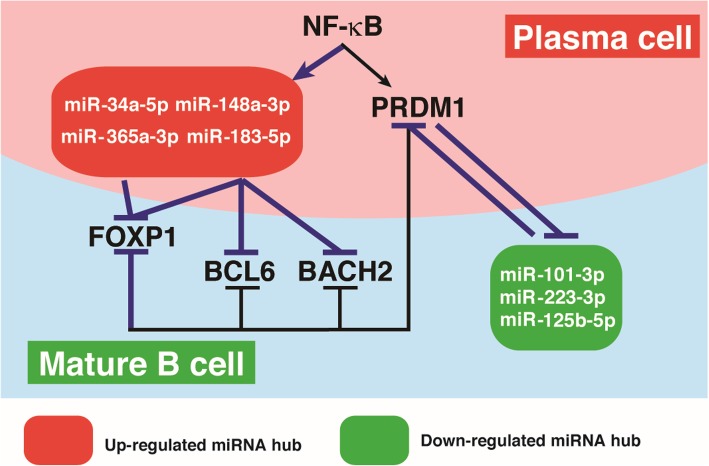
The action of miRNAs and key transcription factors in coordinately directing plasma cell differentiation. Several factors are involved in the negative regulation of *PRDM1* in mature B cells, including *BCL6/BACH2/FOXP1* and the miR-101-3p, miR-125b-5p, miR-223-3p miRNA hub. During B cell activation, NF-κB induces not only *PRDM1* for the initiation of plasma cell differentiation, but also the *miR-34a-5p, miR-148a-3p, miR-183-5p* and *miR-365a-5p* hub. The induced miRNA hub including miR-34a-5p, miR-148a-3p, miR-183-5p and miR-365a-5p downregulates *BCL6/BACH2/FOXP1*, thereby establishing elevated levels of PRDM1 for driving plasma cell differentiation. Induced PRDM1 in turn suppresses the expression of the *miR-101-3p*, *miR-222-3p* and *miR-223-3p* hub, and *BCL6/BACH2/FOXP1*, resulting in commitment to the plasma cell fate. Lines with arrow and bars indicate upregulation and downregulation, respectively. miRNAs in a red or green box represent upregulated or downregulated expression, respectively

We have investigated the changes in the miRNA expression inherent to the transcription network in plasma cell differentiation (Fig. 1) [41]. Two large scale analyses, deep-sequencing and miRNA microarray, were used to elucidate the changes in the expression of miRNAs during human plasma cell differentiation. In this study, human peripheral blood B cells were treated with the stimuli provided by Tfh-mimicking signals. Our computational analysis revealed that 34 and 60 miRNAs with significant reads were upregulated and downregulated, respectively, during human plasma cell differentiation. We characterized the relationship between differentially expressed miRNAs and transcription factors during plasma cell differentiation. We found that several differentially expressed miRNAs commonly target a single key transcription factor. We thus called these miRNAs a “miRNA hub”. It is noteworthy that these miRNA hubs collaboratively regulate the expression of key transcription factors, thereby enabling the formation of human plasma cells in culture. Specifically, we found that upregulated miRNA hubs, including miR-34a-5p, miR-148a-3p, miR-183-5p and miR-365a-3p, directly repressed endogenous *BCL6*, *BACH2* and *FOXP1* expression during plasma cell differentiation. However, downregulated miRNA hubs, including miR-101-3p, miR-125b-5p and miR-223-3p, target the *PRDM1* 3′ untranslated region (UTR). We further showed that NF-κB and PRDM1 contribute to the induction and repression of upregulated and downregulated miRNA hubs, respectively, during plasma cell differentiation. Moreover, our computational analysis unveiled that the transcription factor, FOXP1, is regulated by an induced miRNA hub and plays a role in prohibiting plasma cell differentiation.

With regard to the function of miRNAs in B cells, accumulating reports have demonstrated their roles in regulating B cell development and shaping the properties of the effector functions of B cells. One of the first miRNAs identified with functional significance to B cell development is miR-181a (now called miR-181a-5p). Overexpression of miR-181a-5p in HSCs increased the number of B cells in vitro and in vivo [42]. miR-181a-5p targets the differentiation inhibitor, *ID2*, which suppresses the early differentiation of B cells [43]. MiR-150 regulates the differentiation of normal B cells into antibody-secreting plasma cells. Several studies have indicated that miR-150 is highly expressed in mature B cells, relatively lowly expressed in immature B cells, and has the lowest expression during the pro-B to pre-B cell transition [44, 45]. One key target of miR-150 is *c-Myb*, which is required for the development of pro-B cells [46, 47]. Another study demonstrated that the p53-induced miRNA, miR-34a (now called miR-34a-5p), impaired B cell development. MiR-34a targets the 3’UTR of *Forkhead box protein P1* (*Foxp1*) mRNA, which regulates the expression of recombination-activating genes (*Rag1* and *Rag2*) in the pro-B to pre-B transition stages [48, 49]. MiR-155 is a highly expressed miRNA in GC B cells, however it is expressed at relatively low levels in HSCs and mature B cells [34, 50]. Knockout of the *miR-155* gene in mice caused defective CSR and impaired differentiation of antibody-secreting plasma cells, by targeting *Spi1* (encoding PU.1) and *Aicda* [51–53]. Besides miR-155, miR-181b has been shown to negatively regulate CSR by targeting *Aicda* [54]. Additionally, several other studies have indicated that miR-9, miR-125b, the miR-17–92 cluster and the miR-30 family are expressed in GC B cells and enhance plasma cell differentiation [37, 55]. Deletion of the *miR-17–92* cluster in B cells in mice caused enhanced homing of plasma cells to the bone marrow upon TD immunization, likely owing to the effect of miR-17–92 on *S1pr1*, a gene important for the egress of lymphocytes from the lymphoid organs [56].

### miRNAs in B cell malignancy and autoimmune diseases

Lymphoma, including B and T-cell lymphoma, is malignancy of lymphatic cells, which affects more than a million people worldwide. Many miRNAs that contribute to B cell lymphomagenesis are also key regulators in normal hematopoiesis and lymphopoiesis. MiRNAs that affect tumorigenesis are called onco-miRs or tumor suppressor miRs [57, 58]. The first reported onco-miR is miR-155, which is upregulated in normal plasma cell differentiation and overexpressed in several types of B cell lymphomas [59]. It is noteworthy that mice with miR-155 overexpression in a B cell-specific manner develop high-grade B-cell lymphoma resembling diffuse large B-cell lymphoma (DLBCL) [59], likely owing to the effect of miR-155 on *SHIP1*, which promotes TNFα-dependent cell proliferation [60]. MiR-155 is also a key regulator of the PI3K/AKT pathway in DLBCL. It promotes cell proliferation and inhibits apoptosis of DLBCL cells [61]. The most studied tumor-suppressor miRNA is miR-34a, which forms parts of the p53 network [62]. p53 directly induces miR-34a expression, but at the same time miR-34a enhances p53 expression via inhibiting *SIRT1*, a regulator of p53 deacetylation, resulting in a positive feedback loop [63]. An additional study has shown that miR-34a reduces tumor growth in mice by targeting *Foxp1* [64]. Another well-studied tumor-suppressor miRNA in B cell malignancy is miR-101 (now called miR-101-3p). The decreased expression of miR-101 correlated with the pathogenesis and prognosis of DLBCL, while upregulation of miR-101 in DLBCL inhibited cell proliferation and facilitated apoptosis by targeting *MEK1* [65]. Furthermore, miR-183 is differentially expressed in the three Hodgkin’s lymphoma (HL) subtypes and in EBV^+^ and EBV^−^ HLs. However, elucidation of the exact mode of action of miR-183 in HL requires further investigation [66]. Another study has shown that the expression of miR-223 and miR-125b in DLBCL is higher than in follicular lymphoma (FL), suggesting that the high expression of miR-223 and miR-125b may contribute to the transformation of DLBCL [67]. The aberrant expression of miR-125b in mantle cell lymphoma (MCL) has also been reported. A miRNA expression profile study was able to segregate MCLs into three different groups with distinct biological and clinical features [68].

Unlike DLBCL, which is usually formed from mutated or dysregulated normal GC B cells [69], multiple myeloma (MM) arises from malignant plasma cells in the bone marow [70, 71]. The molecular mechanisms underlying the dysregulation of p53 in MM have been intensively investigated for many years [72, 73]. Recently, it has been suggested that many miRNAs reported to negatively regulate p53 expression may also have implications in MM cells. For example, miR-125b is an onco-miR in hematologic malignancies as it targets *p53* [74] and other components of the p53 pro-apoptotic network, including *BAK1*, *PUMA*, *BMF*, *TRP53INP1* and *Krupple-like factor 13* (*KLF13*) [75, 76]. Furthermore, dexamethasone induces the expression of miR-34a in MM cells [75, 77]. MiR-34a suppresses the expression of SIRT1 deacetylase, and thus allows the maintenance of acetylation and inactivation of p53. Results from a gene microarray study showed that the onco-miRs, miR-19b and miR-20a, were differently expressed in patients with MM and normal controls. MiR19b/20a promotes cell proliferation and migration, and inhibits cell apoptosis by targeting *PTEN* in patients with MM [78]. Moreover, miR-9 is another onco-miR that represses apoptosis in MM patients and MM cell lines by regulating the TRIM56/NF-κB pathway [79]. Taken together, these studies indicate the roles of miRNAs in the progression of B cell malignancy and their therapeutic potential for the treatment of B cell malignancy.

Recently, miRNAs have also been reported to play a role in immune dysfunction and autoimmune diseases. Systemic lupus erythematosus (SLE) is a multisystem autoimmune disorder that mostly affects women at childbearing age [80]. B cells play a cardinal role in the pathogenesis of SLE; more than 90% of SLE patients have high levels of antinuclear antibodies in the sera, including anti-dsDNA antibodies and anti-snRNP antibodies [81]. Several miRNAs have been identified as biomarkers during the development of SLE. miRNA microarray analysis first revealed that miR-21 and miR-17-5p are differently expressed in peripheral blood mononuclear cells (PBMCs) of SLE patients [82]. The increased expression of miR-7 downregulates the expression of PTEN in B cells of SLE patients and contributes to hyper-activation of B cells [83]. Moreover, miR-17-5p is downregulated in PBMCs of SLE patients [82]. MiR-17-5p downregulates c-MYC expression during SLE formation. Transfection of miR-17-5p mimics into PBMCs from SLE patients caused a dramatic reduction in E2F1 and c-MYC expression, which resulted in reduced mRNA levels of the IFN-inducible gene, *MxA* [84]. Screening of SLE-specific miRNAs from 42 B cell-related miRNAs by a miRNA PCR Array identified that 14 miRNAs, including miR-103, miR-150, miR-20a, miR-223, miR-27, miR-15b, miR-16, miR-181a, miR-19b, miR-22, miR-23a, miR-25, miR-92a and miR-93, were significantly downregulated in the plasma of SLE patients, compared with the plasma of healthy donors [85]. Moreover, six plasma miRNAs, including miR-92a, miR-27a, miR-19b, miR-23a, miR-223 and miR-16, were expressed at significantly decreased levels in SLE patients than in rheumatoid arthritis (RA) patients. These results demonstrated that these differentially expressed miRNAs in the plasma of SLE and RA patients can potentially be used as a diagnostic signature to distinguish between these two diseases. Another study using miRNA expression array revealed the serum miRNA expression profiles of SLE patients and healthy donors [86], showing that miR-371b-5p, miR-5100 and miR-146a-5p were increased in active SLE. These studies suggest the potential roles of these identified miRNAs in the development of SLE. The function of miRNAs in B cell malignancy and autoimmune diseases was summarized in Table 1.

**Table 1:** miRNAs in B cell malignancy and autoimmune diseases

miRNA	Biological function	Target genes	Disease type	References
miR-155	Promote TNFα-dependent proliferation	*SHIP1*	DCBCL	[60]
miR-155	Promote proliferation and inhibits apoptosis	PI3K/AKT pathway	DCBCL	[61]
miR-101	Inhibit proliferation and facilitate apoptosis	*MEK1*	DCBCL	[65]
miR-125b	Inhibit apoptosis	*p53*	MM	[74, 75]
miR-19b/20a	Promote proliferation, migration, and inhibit apoptosis	*PTEN*	MM	[78]
miR-34a	Regulate cell cycle progression, cellular senescence and apoptosis	*SIRT1*	MM	[77]
miR-34a	Reduce tumor growth	*Foxp1*	DLBCL	[49]
miR-9	Inhibit apoptosis	TRIM56/NF-κB pathway	MM	[79]
miR-7	Promote B cell hyperresponsiveness	*PTEN*	SLE	[83]
miR-17-5p	Inhibit IFN-inducible gene	*c-MYC,* and *E2F1*	SLE	[84]

### miRNA therapeutics

MiRNA-targeted therapeutics can be divided into miRNA mimics [87] and inhibitors (also called anti-miRs) [88]. The effect of modulation of miRNAs’ levels on B cell malignancy has been demonstrated. Studies on a mouse model of miR-155-induced lymphoma, in which *mir-155* is expressed under the control of doxycycline, demonstrated that doxycycline withdrawal resulted in suppression of *mir-155* expression and subsequent tumor shrinkage [89]. In this mouse model, anti-miR-155 treatment resulted in decreased tumor burden, indicating that miR-155 inhibition has therapeutic potential [89]. In contrast, miR-34a has been identified as a tumor suppressor miRNA by repressing several target genes, such as cyclin-dependent kinase 4 (*CDK4*), *CDK6*, *BCL2*, *MET*, *Notch*, c-*MYC*, *AXL* and *FOXP1* [48, 49, 90]. Several preclinical studies using miR-34 mimics have demonstrated their potential as anticancer therapeutics. For instance, miR-34a mimics showed promising anti-tumor activity in mouse models of lung [91], liver [92] and prostate [93] cancer. In these cases, significant inhibition of tumor growth was observed, which correlated with reduced expression of target proteins, such as c-MYC and BCL-2, in tumors. As a result of the above-mentioned studies, several miRNA-targeted therapeutics have reached clinical development. Currently, there are more than 20 clinical trials applying miRNA and siRNA-based therapeutics [94]. For instance, anti-miRs are single-stranded first-generation antisense oligonucleotides, which have been modified and designed to block the function of miRNAs. Anti-miRs with a 2′-*O*-methoxyethyl modification (2′-OM) are called antagomiRs [95]. These synthetic small RNA molecules have a complementary sequence to the target miRNA, and are able to strongly bind to the target miRNA and thereby block its function. MiRNA mimics are synthetic double-stranded small RNA molecules matching the corresponding miRNA sequence, and therefore functionally able to restore the loss of miRNA expression in diseases. MiR-34a mimics reached phase I clinical trials for treating cancer, but this trial was halted at phase I owing to immune-related adverse events [94]. Effective delivery of RNA-based therapeutics to the target tissues has been a challenge in their therapeutic application [96]. Development of better in vivo delivery systems to reach the target specifically and efficiently to overcome the bottleneck of RNA-based therapy (including miRNA) in the clinic is the next important task.

### Epigenetic regulation in B cell activation and differentiation

Epigenetic regulation is critical for coordination with the abovementioned transcription regulation networks in molecular programming during B cell activation and differentiation. The synergistic effects of both genetically and environmentally induced epigenetic modifications have been demonstrated to contribute to plasma cell differentiation and the etiopathogenetic mechanisms of the generation of B cell- or plasma cell-related diseases, such as autoimmune disorders and lymphomagenesis [97]. In general, the quiescent naïve B cells in peripheral lymphoid organs display inactive chromatin structures that show genome-wide DNA hypermethylation [98] and methylation of histone 3 K9 (H3K9) and H3K27 [99]. At this stage, the expression level of genes important for regulating B cell identify and antigen recognition is regulated by histone deacetylase 7 (HDAC7) [100]. During early B cell development, HDAC7 represses myeloid and T cell genes in early B cell progenitors [100]. Enhancer of zeste homolog 2 (Ezh2) is able to catalyze H3K27me3, which is associated with long-term repression [101]. In GC B cells, Ezh2 is highly expressed [102]. Deletion of *Ezh2* in mice in a GC-specific manner caused impaired GC response, memory B cell formation and antibody responses compared with the control mice, suggesting that Ezh2 is essential for B cell functions [103]. In GC reactions, a number of histone modifications, including acetylated H3 and H4, and DNA double-strand breaks (DSBs)-induced phosphorylated H2AX (γH2AX), are associated with CS [104, 105]. However, acetylated H3 and H4 may not be linked with SHM activation. Instead, the histone modification pattern of SHM consists of phosphorylation of histone H2B on serine 14 (H2B^Ser14P^), which is also responsive to DSBs [106].

In addition, in GC, the expression and action of AID is regulated by a series of epigenetic mechanisms. The suppression of *Aicda* in naïve B cells is due to DNA hypermethylation at the promoter region [107]. The H3 acetylation level of the *Aicda* gene locus in naïve B cells is low compared with the global H3 acetylation levels of other nearby genes. After B cells are stimulated, the *Aicda* gene locus is demethylated and becomes enriched with H3K4me3, H3K9ac and H3K14ac, which are associated with active histone marks [108]. Downregulation of *Aicda* in memory B cells and plasma cells may result from re-methylation of the *Aicda* gene locus. The histone chaperone, Spt6, regulates CSR and AID expression through two distinct types of histone modifications to generate the euchromatin status, namely, H3K4me3 and H3K36me3, respectively. Spt6 is also required for the establishment of H3K4me3 marks in the IgH variable region during SHM [109]. In terms of the functional mode of action of AID, it interacts with ubiquitinated chromatin. Specifically, ubiquitination of H2BK120 and H2AK119 is colocalized with mismatched DNA polymerase η in the AID-containing region [110].

**Fig. 2:**
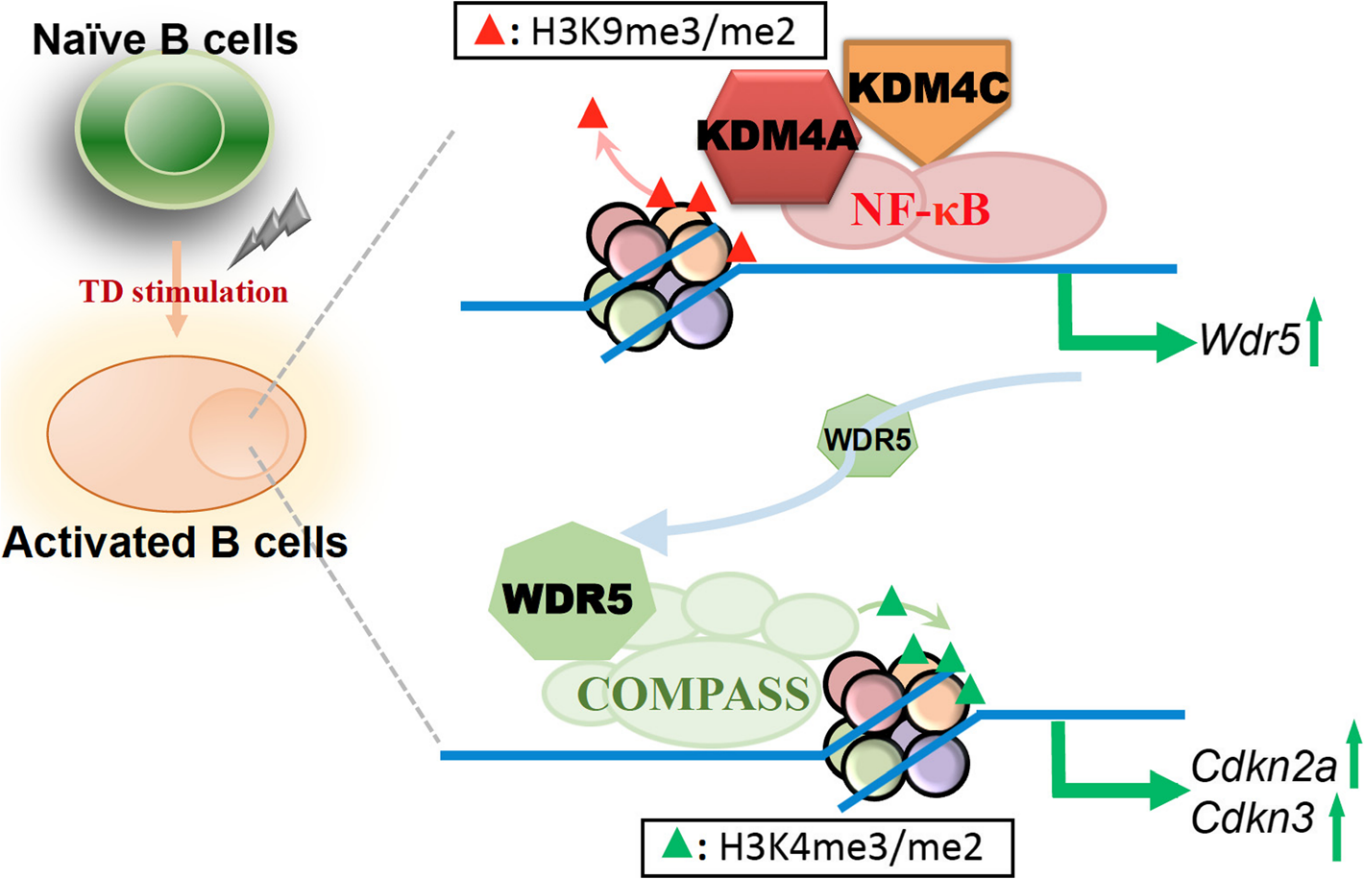
Proposed model of the role of KDM4A and KDM4C in B cell activation. During activation by Tfh cell-mimicking signals, the induced demethylases, KDM4A and KDM4C, cooperate with NF-κB to upregulate the expression of *Wdr5* by removing H3K9me3/me2. WDR5, a core subunit of the COMPASS histone H3K4 methyltransferase complex, in turn facilitates the transcription of *Cdkn2a* and *Cdkn3* by elevating H3K4me3/me2. Both CDKN2A and CDKN3 are involved in the regulation of stimulated B cell proliferation

The global levels of H3K9me2/me3 and H3K4me2 are all upregulated after LPS and IL-4 stimulation in a B cell culture [111]. We have also reported changes in histone modifications in B cells treated with Tfh cell-mimicking signals (Fig. 2) [99]. We found that the global levels of H3K9me3/me2 were reduced after stimulating mouse spleen B cells with Tfh cell-mediated signals. Furthermore, a systemic search of the epigenetic modifiers that contribute to the downregulation of H3K9me3/me2 revealed that the histone demethylases, KDM4A and KDM4C, were upregulated in mouse spleen B cells treated with Tfh cell-mimicking signals, whereas stimulation with LPS did not induce a similar pattern of KDM4A/KDM4C-mediated epigenetic changes. Functionally, depletion of KDM4A and KDM4C in response to Tfh cell-mimicking signals accelerated B cell activation and proliferation. Our genome-wide analysis using chromatin immunoprecipitation sequencing (ChIP-seq) combined with cDNA microarray analyses further revealed KDM4A and KDM4C targets during B cell activation. Among these, WDR5, an MLL complex member that facilitates H3K4 methylation [112], was further demonstrated to regulate the cell cycle; in particular, the cell cycle inhibitors, *Cdkn2* and *Cdkn3*. Mechanistically, de novo motif analysis of the ChIP-seq data of KDM4A and KDM4C revealed that NF-κB p65 interacts with KDM4A and KDM4C to regulate gene expression, including *WDR5*.

Less is known about epigenetic modification during plasma cell differentiation. Genes expressed during plasma cell differentiation correlated with the acquisition of H3K4me1 and H3K4me3 histone marks, which are the markers of active promoters and distal enhancers [113]. Blimp-1 is a key transcription factor in directing plasma cell differentiation [15], thus, epigenetic regulation of *PRDM1* expression and function would affect the humoral responses. BCL6 and HDAC4, − 5 or − 7 form stable complexes to mediate the decreased levels of histone acetylation on the *PRDM1* promoter in GC B cells [114, 115]. Furthermore, treatment of B cells with HDAC inhibitors, such as trichostatin A (TSA) and butyrate, induced the expression of Blimp-1 and J chain, but decreased the expression of c-Myc and Pax-5. TSA treatment also promotes the expression of CD138 and downregulates surface IgM [116]. Several Blimp-1 target genes, including *Pax5* and *Spib*, showed decreased histone acetylation in plasma cells [117, 118]. Therefore, histone acetylation regulates the transcription of genes controlling B cell differentiation. In general, Blimp-1 is a transcription repressor able to recruit co-repressor proteins and histone modifiers to induce transcription repression. We have previously shown that a proline-rich domain of Blimp-1 directly interacts with LSD1, an H3K4me2/1 and H3K9me2/1-specific demethylase [117]. These proteins collaboratively remodel the chromatin accessibility of Blimp-1 targets, and thereby modulate the expression of Blimp-1 targets [117]. Disruption of the Blimp-1 interaction with LSD1, depletion of LSD1 expression and inhibition of LSD1 function all reduced the formation of antibody-secreting plasma cells. The five C2H2 zinc fingers of Blimp-1 contain a nuclear localization signal and show DNA binding activity. They interact with HDAC2 [118] and H3K9-specific methyltransferase G9a [12], both of which are histone modifiers that facilitate the inactive chromatin and reduce transcription. These findings suggest that Blimp-1 regulates the differentiation of plasma cells by interacting with multiple chromatin modifiers.

### Dysregulated epigenetic mechanisms in B cell malignancy and autoimmune diseases

The dysregulated epigenetic mechanism resulting in inadequate cell cycle is one of the major causes leading to malignant B cells. For instance, the H3K27-specific methyltransferase EZH2 is highly expressed in the GC, where it cooperates with Polycomb Repressive Complex-2 (PRC2) [119]. Mutation of *Ezh2* in mice resulted in GC hyperplasia [120], which in part may stem from the enhanced repression of *CDKN1A* [120]. Furthermore, EZH2 can restrain plasma cell differentiation by establishing the H3K27me3 marks at the *Prdm1* and *Irf4* loci [103]. In human DLBCL cells, a mutant form of EZH2 silences *PRDM1* through the recruitment of PRC2, resulting in the promotion of growth of the GC-type DLBCL [103]. Hence, 22% of GC-type DLBCL cases carry EZH2 mutations [121]. In addition, KMT2D (also known as MLL2 or MLL4) is a member of the SET1 family of histone methyltransferases (HMTs) that facilitates the establishment of H3K4me2/me1 at enhancer regions and was frequently found to be mutated in FL (accounting for 70–80% of cases) and DLBCL [122–124]. KMT2D regulates genes involved in the CD40, JAK-STAT, TLR and BCR signaling pathways [123]. Mice harboring a *Kmt2d* deletion in B cells showed B cell proliferation advantages and B cell lymphoproliferative diseases through collaboration with BCL-2 [124]. The *CREBBP* gene encodes a H3K18 and H3K27-specific lysine acetyltransferase that tags the transcription activation [125, 126]. Further inactivating mutations and/or copy number losses of *CREBBP* occur in more than half of the FL cases and in nearly 20% of the DLBCL cases [127]. Accordingly, *Crebbp* deletion in the GC stage in mice in the presence of BCL-2 overexpression promotes the development of FL [128].

MM is plasma-cell malignancy with a slow progress feature [129]. One of the well studied HMTs in MM is the H3K36me2 and H4K20me2/me3-specific lysine methyltransferase, MMSET (also known as WHSC1 or NSD2) [130, 131]. MMEST is a DNA damage-responsive protein that catalyzes H4K20 methylation and induces the accumulation of 53BP1 at DNA damage sites [132]. Fifteen to 20 % of MM cells carry the t(4;14) translocation, which generates the fusion of MMSET to the *IgH* locus, resulting in the upregulation MMSET, which correlates with poor prognosis [133]. MMSET forms a complex with epigenetic repressors by recruiting Sin3a, HDAC1, HDAC2 and the H3K4-specific demethylase, LSD1/KDM1A. The MMSET complex then indirectly induces c-MYC levels by repressing miR-126, thereby sustaining the proliferation of MM cells [134, 135]. Nevertheless, the t [4, 14] translocation in MM cells remodels the chromatin structure that carries the H3K36me2 histone activation mark and induces global reduction in H3K27me3 by upregulating EZH2 at the oncogenic loci [131, 133]. The extent of H3K27 methylation is associated with the malignancy of plasma cells. EZH2 is upregulated during MM progression [136], resulting in enhanced IL-6R expression, c-MYC activation, miR-26a downregulation and long non-coding RNA expression, thereby affecting the proliferation and apoptosis of MM cells [137, 138]. In contrast, mutations of the H3K27-specific demethylase, KDM6A, are found in 10% of primary MM samples [138, 139]. Inhibition of EZH2 decreases the growth of KDM6A-depleted MM cells. The KDM6A-mutated MM cells are more sensitive to EZH2 inhibitor-induced apoptosis through reactivation of BCL6 and subsequently repression of *IRF4* and *c-MYC* [140]. These results showed the synergetic effect of EZH2 and KDM6A, which collaboratively control the expression of a set of oncogenic genes. The levels of the H3K9-specific demethylase, KDM3A, have been demonstrated to be increased in MM cells. It has been shown that the KDM3A-KLF2-IRF4 axis promotes homing of MM cells to the bone marrow and their adherence to bone marrow stromal cells. KDM3A maintains the transcriptional activity of *KLF2* and *IRF4* [141, 142]. KDM3A is upregulated by hypoxia-induced HIF1α that induces the expression of the long non-coding RNA, *MALAT1*, which in turn facilitates the upregulation of glycolytic and anti-apoptotic genes in MM cells [143, 144].

Recent reports have shown the significance of abnormal epigenetic regulation in the pathogenesis of SLE. A significantly reduced level of *DNMT1* and *DNMT3A* transcripts was found in SLE patients compared with healthy controls [145]. The high IL-6 levels produced by SLE patients resulted in impaired induction of DNMT1, which in turn caused the demethylation of DNA in CpG islands in the cytoplasmic isoform of *CD5*, *CD5-E1B*. CD5-E1B is a negative regulator of BCR signaling, thereby establishing the immune tolerance in SLE B cells [146, 147]. Beyond the coding genes, the DNA methylation state of non-coding regions in the genome of SLE patients was also found to be altered. The hypomethylated long interspersed nuclear elements, but not the short interspersed nuclear elements, in SLE B cells correlate with the disease prognosis [148, 149]. Furthermore, our previously identified KDM4A/KDM4C/WDR5/CDKNs epigenetic pathway induced by Tfh cell-mimicking signals is dysregulated in B cells isolated from SLE patients [99]. We found that activated normal human peripheral blood B cells exhibited a significant reduction in H3K9me2 and H3K9me3, while the levels of H3K9me2 and H3K9me3 in stimulated SLE B cells did not change significantly. Accordingly, *KDM4A* and *KDM4C* mRNA levels were significantly reduced in the steady state and the stimulated SLE B cells, compared with normal B cells. Together, the effects of dysregulated histone modifiers on B cell malignancy and autoimmune diseases were summarized in Table 2.

**Table 2:** Epigenetic controls in B cell malignancy and autoimmune diseases

Histone Modifier	Biological function	Target genes *(direct or indirect)*	Disease type	References
EZH2	Promote growth	*CDKN1A*	DCBCL	[120]
EZH2	Retrain plasma cell differentiation	*PRDM1* and *IRF4*	Non-Hodgkin lymphomas	[103]
KDM4A	Promote B cell activation and differentiation	*WDR5*	SLE	[99]
KDM4C	Promote B cell activation and differentiation	*WDR5*	SLE	[99]
LSD1	Promote differentiation	Blimp-1 targets, such as *CIITA* and *c-MYC*	MM	[117]
MMSET	Promote growth	*c-MYC*	MM	[135]
KDM6A	Promote oncogenesis	*BCL-6*, *IRF4* and *c-MYC*	MM	[140]
KDM3A	Promote homing of MM cells	*KLF2*, and *IRF4*	MM	[141]
WDR5	Inhibit proliferation	*CDKN2,* and *CDKN3*	SLE	[99]
CREBBP	Regulate GC reaction and proliferation	BCL-6 targets, such as *BCL-2* and *PRDM1*	FL and DCBCL	[128]
KMT2D	Repress lymphoma development	Genes in CD40, JAK-STAT, TLR and BCR pathways	FL and DCBCL	[123]
DNMT1	Promote BCR signaling	CD5, and CD5-E1B	SLE	[146]

### Epigenetic therapeutics

The aberrant epigenetic profiles of malignant cells, such as in MM, have been established in past decade. To target these aberrant epigenetic regulation mechanisms in MM, there are three categories of epigenetic modulating therapeutic agents under development: DNA methyltransferase inhibitors (DNMTi), histone deacetylase inhibitors (HDACi) and histone lysine methyltransferase inhibitor (HKMTi). Several DNMTis, such as 5-azacytidine (AZA, Vidaza) and 2-deoxy-5-aza-cytidine (DAC, Decitabine, Dacogen), have been ideal therapeutics for myelodysplastic syndrome [150, 151]. In MM, AZA and DAC have been demonstrated to have anti-MM effects, which cause cell cycle arrest and generation of oxidative stress to induce necrosis and apoptosis [152, 153]. In recent years, great progress has been achieved with HDACis in drug development for cancer therapy. The anti-MM effects of HDACis rely on their chromatin remodeling activity to induce apoptosis, cell cycle arrest and autophagy, as well as to suppress angiogenesis [154]. In particular, HDAC6 regulates deacetylation of α-tubulin and heat shock protein 90α (HSP90α), thereby affecting cell motility and cell adhesion, as well as the aggresome degradation pathway in response to misfolded proteins in MM cells [155]. Furthermore, inhibition of HDAC6 shows minimal side effects on healthy cells [156]. Hence, several clinical trials have applied HDAC6-specific inhibitors, such as Rocilinostat [157], ITF2357 [158] and Panobinostat [159, 160], in MM therapy.

The bromodomain (BRD) and extra-terminal (BET) family of BRD-containing proteins is a group of proteins that recognize acetylated lysine residues of histones and regulate gene expression. Hence, suppression of the activity of BRD-containing proteins is an effective way to control the histone-acetylation-dependent gene activation. The pan-BET inhibitor, GSK525762, inhibits growth of a broad spectrum of human hematological cancer cells, including MM [161]. Other ongoing clinical studies showed that BET inhibitors, such as OTX015 and CPI-0610, which selectively block BRD2, BRD3 and BRD4, were also used in the pre-clinical or clinical trials for MM or lymphoma [162, 163].

In addition, EZH2 inhibitors have been developed to block MM. Currently, the EZH2 inhibitor, Tazemetostat (EPZ-6438), is under clinical trials in combination with immunomodulatory imide drugs (IMiDs) for treating a subgroup of MM patients [164]. GSK2816126, another EZH2 inhibitor, is also in clinical trials for MM. It induces apoptosis in MM cells by downregulating mitochondrial activity [165].

## Conclusions

Accumulating research efforts have been made to elucidate the molecular pathways regulating the B cell responses and antibody production. Studying the regulatory mechanisms of B cell responses has become an emerging research topic with the need to further understand the pathways that control the new coming pathogens through vaccination or to combat cancers. In addition to the above-described regulatory mechanisms in B cell activation and differentiation, there are other types of regulation involved, such as glycosylation and SUMOylation. Studies on these regulatory mechanisms open opportunities for identifying new druggable targets to control B cell-related diseases such as autoimmune diseases and B cell malignancies.

## Data Availability

Not applicable.

## Abbreviations

2′-OM: 2′-*O*-methoxyethyl
AID: Activation induced cystidine deaminase
ASOs: Antisense oligonucleotides
BACH2: BTB domain and CNC homologue 2
BCL6: B-cell lymphoma 6
BCRs: B cell receptors
BET: Bromodomain extra-terminal
Blimp-1: B lymphocyte-induced maturation protein-1
BRD: Bromodomain
CDK4: Cyclin-dependent kinase 4
CDK6: Cyclin-dependent kinase 6
CLPs: Common lymphoid progenitors
CSR: Class switch recombination
DLBCL: Diffuse large B-cell lymphoma
DNMTi: DNA methyltransferase inhibitors
EBF: Early B-cell factor
ER: Endoplasmic reticulum
Ezh2: Enhancer of zeste homolog 2
FDCs: Follicular dendritic cells
FL: Follicular lymphoma
FOXP1: Forkhead box protein P1
GC: Germinal center
H3K27: methylation of histone 3 K27
H3K9: methylation of histone 3 K9
HDAC7: Histone deacetylase 7
HDACi: Histone deacetylase inhibitor
HKMTi: Histone lysine methyltransferase inhibitor
HL: Hodgkin’s lymphoma
HMTs: Histone methyltransferases
HSCs: Hematopoietic stem cells
IKZF3: Ikaros family zinc finger protein 3
IMiDs: Immunomodulatory imide drugs
IRF4: Interferon-regulatory factor 4
IRF8: Interferon-regulatory factor 8
KLF13: Krupple-like factor 13
LPS: Lipopolysaccharides
MCL: Mantle cell lymphoma
miRNA: microRNA
MM: Multiple myeloma
MMSET: H4K20me2/me3-specific lysine methyltransferase
PAX5: Paired box gene 5
PBMCs: Peripheral blood mononuclear cells
PRC2: Polycomb Repressive Complex-2
PRDM1: PR domain zinc finger protein 1
RA: Rheumatoid arthritis
RAG-1: Recombination-activating gene-1
RAG-2: Recombination-activating gene-2
SHM: Somatic hypermutation
SLE: Systemic lupus erythematosus
TD: T-cell dependent
Tfh: follicular helper T
TI: T-cell independent
TSA: Trichostatin A
XBP-1: X-box-binding protein 1
